# Hyponatraemia as a Predictor of Mortality in Medical Admissions in Ghana: A Comparative Study

**DOI:** 10.1155/2020/3145843

**Published:** 2020-11-22

**Authors:** Elliot Koranteng Tannor, Martin Agyei, Abena Y. Tannor, Afua Ofori, Emmanuel Akumiah, Yasmin Adoma Boateng

**Affiliations:** ^1^Renal Unit, Department of Medicine, Komfo Anokye Teaching Hospital, Kumasi, Ghana; ^2^Department of Internal Medicine, School of Medicine and Dentistry, Kwame Nkrumah University of Science and Technology, Kumasi, Ghana; ^3^Department of Family Medicine, Komfo Anokye Teaching Hospital, Kumasi, Ghana; ^4^Research and Development Unit, Komfo Anokye Teaching Hospital, Kumasi, Ghana

## Abstract

**Background:**

Hyponatraemia is the most common electrolyte abnormality in hospital admissions. It occurs in a quarter of medical admissions in Ghana, and it is associated with high mortality. Mortality has been suggested to be due to the underlying medical condition and not necessarily the hyponatraemia. We set out to compare the outcomes of patients with documented hyponatraemia as compared to those with normonatraemia in terms of mortality and length of hospital stay.

**Methods:**

We conducted a comparative analysis of patients with hyponatraemia and those with normonatraemia on the medical ward at the Komfo Anokye Teaching Hospital between May 2018 and December 2018. The medical diagnoses, demographics, and laboratory data of the patients were recorded. Participants' age and gender were matched. Student's *t*-test was used to test for differences in continuous variables when parametric and Wilcoxon signed-rank test for nonparametric variables. Multiple logistic regression was used to identify predictors of in-hospital mortality. A *p* value of <0.05 was considered statistically significant.

**Results:**

Within the study period, 846 patients with documented serum sodium were included in the study. The study involved 406 patients with hyponatraemia and 440 patients with normonatraemia. Serum albumin and protein were significantly lower in the hyponatraemia patients as compared to those with normonatraemia. The mortality rate in patients with hyponatraemia was significantly higher than those with normonatraemia (129 (31.8%) vs. 9 (22.3%); OR 1.62 (95% CI: 1.19–2.22), *p* = 0.002). In-hospital stay was longer in patients with hyponatraemia than normonatraemia (7 (4–10) vs. 6 (3–10) days) but not statistically significant (*p* = 0.09). Multiple logistic regression showed that low serum sodium (*p* < 0.001) and low serum albumin (*p* = 0.009) were the predictors of in-hospital mortality.

**Conclusion:**

Hyponatraemia is associated with significantly higher mortality than normonatraemia and predicts worse prognosis in patients on medical admission. Low serum albumin is also a predictor of mortality in medical admission.

## 1. Background

Hyponatraemia is the most common electrolyte abnormality in hospitalized patients [1]. It is defined as a serum sodium concentration of less than 135 mmol/L [2]. Hyponatraemia occurs in 27.6% of hospitalized medical patients in Ghana [3] and associated with high mortality [4]. Hyponatraemia indicates a disruption in the body's water balance due to abnormal handling of water by the kidneys from antidiuretic hormone (ADH) secretion (appropriately or inappropriately) or an excessive water intake [2, 5]. There are multiple causes of hyponatraemia among medical admissions. These include the use of some medications, malignancies, infections, chronic heart failure, chronic liver disease, and chronic kidney disease [3]. Hyponatraemia is most commonly seen in patients admitted in the intensive care unit [6]. Nonosmotic stimuli of ADH secretion such as pain, anxiety, and nausea are also reported as causes of hyponatraemia in acute cases [7]. Infections have been shown to be the most common cause of hyponatraemia among medical admissions in Ghana [3].

Hyponatraemia is associated with poor prognosis and mortality in chronic hospitalized medical cases even when mild to moderate [4, 8]. Hyponatraemia was associated with mortality rate ranging from 0.9 to 29.6% in hospital medical admissions. The highest mortality rates are seen among patients in the intensive care unit [6, 9]. The in-hospital mortality in medical patients was found to be 31.8% in a recent hospital-based study in Ghana [3]. It is however not certain whether hyponatraemia is direct cause of mortality, a poor prognostic indicator for chronic medical conditions such as malignancies, chronic kidney disease, chronic liver disease, brain tumour, and intracerebral hemorrhage, [4] or whether mortality is due to the underlying condition and not necessarily the hyponatraemia [10].

It has been suggested that most patients die with hyponatraemia rather than from hyponatraemia [11] with the underlying medical condition as the most likely cause of death. This is because mortality varies with the underlying condition. It has been shown that drug-induced hyponatraemia is associated with less mortality [11] while almost half of patients with malignancy die with hyponatraemia [3].

In Ghana, there has been a single-centre study to describe the causes and outcomes of patients with hyponatraemia in medical admissions [3], but there has not been any study to compare the mortality of patients with hyponatraemia and those with normal sodium concentration in hospitalized patients to ascertain if there is a difference in mortality and length of hospital stay. With our knowledge of the mortality in patients with hyponatraemia, we set out as our primary outcome to compare the in-hospital mortality and length of hospital stay of patients with documented hyponatraemia to patients with normonatraemia. Our secondary outcome was to determine the predictors of in-hospital mortality among medical admissions in tertiary institution in Ghana.

## 2. Methods

We conducted a comparative analysis of patients with hyponatraemia and compared to those with normonatraemia on the medical ward at the Komfo Anokye Teaching Hospital (KATH). The study was conducted between May 2018 and December 2018 as a follow-up to an earlier study of patients with only hyponatraemia conducted from October 2017 to April 2018 [3]. We identified medical patients on admission with age greater than 18 years and above with documented normonatraemia and recorded their medical diagnoses, demographics, and laboratory data from their clinical notes. Participants with normonatraemia were age and gender matched as much as possible with those with hyponatraemia.

Data were collected from medical records of patients on admission into the renal registry database at the KATH. KATH is a 1200-bed hospital in the Ashanti region. KATH receives referrals from about half of Ghana's estimated population of about 30 million people [12] and serves the northern half of the country. The hospital has twelve (12) clinical directorates including the internal medicine directorate which has a bed capacity of 203. Ethical approval was granted before the commencement of the study by the Committee on Human Research, Publications and Ethics (CHRPE), School of Medicine and Dentistry, Kwame Nkrumah University of Science and Technology (KNUST).

### 2.1. Study Participants

Patients with recorded serum concentration were divided into two arms if they were 18 years and above. Hyponatraemia was defined as a serum sodium concentration of less than 135 mmol/L [2]. Those patients with recorded serum sodium concentration above 135 mmol/L but less than 146 mmol/L were grouped into the normonatraemia arm as controls. Demographic data including age and gender were recorded and matched as much as possible. Laboratory investigations including serum albumin, serum protein, serum creatinine, and serum urea concentrations were also documented. The medical diagnoses in patients' folder were also recorded, and the most likely cause of hyponatraemia as per the clinical and laboratory parameters was then established by the nephrologist or as stated in the patients' medical records. If more than one cause of hyponatraemia was identified, the predominant diagnosis was chosen as the cause of hyponatraemia according to the nephrologist's discretion. The medical diagnoses of those with normonatraemia were also recorded and classified broadly as those for hyponatraemia to aid analysis. Outcome variables documented were the length of hospital stay in days and in-hospital mortality. Length of hospital stay refers to the duration of stay from admission to discharge of the patient from the medical ward, and in-hospital mortality was defined as death from any cause occurring during hospital admission.

### 2.2. Statistical Analysis

Data were collected onto a data capturing sheet and entered into Microsoft Excel software by two independent data entry clerks. Data were then exported to Stata 13® statistical software for analysis. Means and standard deviation were used to describe parametric variables and medians and interquartile range for nonparametric variables. Tables were used to describe and represent data. Chi square was used to test for significant differences in categorical variables for hyponatraemia and normonatraemia arms, and odds ratios were determined. Student's *t*-test was used to test for differences in continuous variables when normally distributed involving two means, and the Wilcoxon signed-rank test was used for continuous variables when not normally distributed for the two arms. Multiple logistic regression was then used to identify predictors of mortality, and a *p* value of <0.05 was considered statistically significant.

## 3. Results

The study involved 846 hospitalized patients on the medical ward of the Komfo Anokye Teaching Hospital. They included 406 patients with hyponatraemia as cases and 440 patients with normonatraemia as controls. The mean age of all participants was 52.1 ± 18.8 years, and there were 460 (54.4%) males. There was no statistical difference in demographic data with respect to age and gender between the patients with hyponatraemia and those with normonatraemia, as shown in Table 1.

The most common medical conditions associated with hyponatraemia were infections in 105 (25.9%), chronic liver disease in 69 (17.0%), hyperglycaemia in 68 (16.8%), and chronic kidney disease in 66 (16.3%). The most common medical conditions in the normonatraemia group were intracranial pathologies in 131 (29.8%), chronic heart failure in 76 (17.3%), and infections in 72 (16.4%), as shown in Table 1.

Infections, chronic kidney disease, liver disease, and hyperglycaemia were significantly of higher proportions in patients with hyponatraemia as compared to patients with normonatraemia. Malignancies and alcoholism did not vary significantly within the two groups. There were lower proportions of patients with heart failure and cranial pathologies in the hyponatraemia group as compared to those with normonatraemia.

In-hospital mortality was significantly higher in patients with hyponatraemia as compared to patients with normonatraemia (129 (31.8%) vs. 98 (22.3%); *p* = 0.002), as shown in Table 1.

Hyponatraemia was associated with increased odds of in-patient mortality (OR 1.62 (95% CI: 1.18–2.22); *p* = 0.002). Hypoalbuminaemia and hyperglycaemia were also identified as significantly predictive of in-hospital mortality, as shown in Table 2.

Multiple logistic regression shows that serum sodium concentration (OR = 0.94 (95% CI: 0.92–0.97); *p* < 0.001) and serum albumin concentration (OR = 0.97 (95% CI: 0.94–0.99); *p* = 0.007) were independently predictive of in-hospital mortality, as shown in Table 3.

## 4. Discussion

This is the first study according to our knowledge that compares patients with hyponatraemia with those with normonatraemia in Ghana. We found out that patients with hyponatraemia had significantly higher in-hospital mortality as compared to those with normonatraemia. Our study adds to the growing evidence of the significant role of hyponatraemia in predicting in-hospital mortality among medical admissions [4, 8, 9] and more importantly has normonatraemia as a control group. We highlight the need to identify, monitor, and appropriately manage patients with hyponatraemia in medical admissions.

We found patients with hyponatraemia to have significantly higher proportions of chronic kidney disease, hyperglycaemia, and chronic liver disease than those with normonatraemia. These medical conditions have also been shown in other studies as associations and notable causes of hyponatraemia [3, 13, 14]. Hyponatraemia has been shown to be a poor prognostic indicator in chronic medical conditions such as chronic liver disease [15], chronic heart failure [16], and chronic kidney disease [17]. Mortality in chronic medical conditions has been shown to be predicted by the serum sodium concentration [8, 9, 17], as also found in our study in Ghana.

With a high prevalence of hyponatraemia in medical admissions in a previous study [3], it becomes even more clear as shown by our study the need for clinicians to pay more attention to hyponatraemia in the management of chronic and acute medical conditions to improve the survival of their patients.

There is evidence to show that hyponatraemia can directly cause death in acute hyponatraemia as a result of cerebral oedema or rapid correction of chronic hyponatraemia leading to osmotic demyelination syndrome [5, 18], but osmotic demyelination syndrome is rarely reported as a cause of death in patients with hyponatraemia [3]. Osmotic demyelination syndrome has been shown to occur more commonly in alcoholics, malnourished, and hypokalaemic patients with chronic hyponatraemia [19], especially when rapidly corrected. Such patients should be managed with a lot of caution to avoid osmotic demyelination syndrome with slow and careful correction when they report with chronic hyponatraemia.

Hyponatraemia may cause organ dysfunction which may also lead to mortality. Hyponatraemia can cause falls, osteoporosis, and fractures [20], but there has not been any proof of effect on other organs such as the heart, liver, and kidneys. Hyponatraemia has also been shown to be an independent predictor of mortality in various studies [4, 8, 9, 21], but not many studies have been conducted in sub-Saharan Africa and hence this study.

A major confounder identified in our study as an independent predictor of in-hospital mortality was hypoalbuminaemia. Hypoalbuminaemia has also been shown to be an independent predictor of mortality in hospitalized patients in general and in conditions such as chronic kidney disease and strokes [22–25]. In our study, those with hyponatraemia had significantly lower serum albumin as compared to those with normonatraemia (31.6 ± 16.5 vs. 36.1 ± 22.4; *p* < 0.001) and may have accounted independently to increase the mortality in the hyponatraemia arm of the study.

Serum albumin is a major component of plasma protein, and it is required to maintain oncotic pressure and also serves as a means of assessing nutritional status in both acute and chronically ill patients [26]. The levels of serum albumin may decrease as a result of many factors. These include poor nutritional status and chronic inflammation as a result of the underlying chronic disease causing hyponatraemia such as infection or malignancy due to interleukin-1 and tumour necrosis factor which decrease hepatic albumin production. Albumin could also be lost via the kidneys in glomerular diseases or during burns as well as in high metabolic and catabolic states [22]. Hypoalbuminaemia has been associated with increased morbidity and mortality in hospitalized patients, but the role of hypoalbuminaemia directly on mortality is still debated [22–25].

We have also shown that hyponatraemia is an independent predictor of mortality but not necessarily a direct cause of mortality. Looking out for hyponatraemia may help prognosticate chronic medical conditions and to avoid rapid correction to prevent osmotic demyelination syndrome especially in those with hypokalaemia, alcoholics, and chronic malnutrition who present with chronic hyponatraemia [19]. Routine serum sodium measurements may help decrease mortality in patients with chronic medical conditions and appropriately manage them.

Our study had a number of limitations. It was a retrospective study, and we reported only those with documented laboratory parameters in their clinical notes. The diagnoses were those made by the attending physicians, and those with no clear diagnosis for the hyponatraemia were reviewed by the nephrologist for diagnosis based on the available data which may be prone to bias. The authors therefore recommend a prospective randomized control trial to establish the risk of mortality with hyponatraemia and match other confounders such as the clinical diagnoses, serum albumin, and serum protein and to exclude patients with other comorbidities which may confound as independent predictors of mortality.

## 5. Conclusion

The in-hospital mortality of patients with hyponatraemia is significantly higher than those with normonatraemia. Hyponatraemia predicts worse prognosis in patients on medical admission irrespective of their underlying diagnosis. Hypoalbuminaemia is also an independent predictor of in-hospital mortality among medical admissions.

## Figures and Tables

**Table 1:** Comparison of patients with hyponatraemia and normonatraemia (*n* = 846).

Variable	All cases, *N* = 846	Hyponatraemia, *N* = 406	Controls, *N* = 440	*P* value
Male gender, *n* (%)	460 (54.4)	217 (53.5)	243 (55.2)	0.604
Age (years), *μ* (SD)	52.1 ± 18.8	51.5 ± 19.0	52.7 ± 18.6	0.340
Serum sodium (mmol/L), *μ* (SD)	133.8 ± 7.6	128 ± 7.8	138.0 ± 5.0	<0.001
Serum albumin (g/L), *μ* (SD	33.9 ± 19.8	31.6 ± 16.5	36.1 ± 22.4	<0.001
Serum protein (g/L), *M* (IQR)	66.5 (58–75.6)	64 (55.2–75.6)	71 (63.3–78.8)	<0.001
Serum urea (mmol/L), *M* (IQR)	5.8 (3.44–12.0)	6.9 (3.9–15.8)	5.2 (3.3–9.6)	<0.001
Serum creatinine (*μ*mol/L), *M* (IQR)	90 (61.1–170)	95 (61–232.5)	88 (62–144)	0.091
Medical diagnosis Infections, *n* (%)				
	177 (20.9)	105 (25.9)	72 (16.4)	0.001
Malignancies, *n* (%)	22 (2.6)	11 (2.7)	11 (2.5)	0.848
Heart failure, *n* (%)	109 (12.9)	33 (8.1)	76 (17.3)	<0.001
Cranial pathologies, *n* (%)	154 (18.2)	23 (5.7)	131 (29.8)	<0.001
Chronic kidney disease, *n* (%)	96 (11.4)	66 (16.3)	30 (6.8)	<0.001
Hyperglycaemia, *n* (%)	98 (11.6)	68 (16.8)	30 (6.8)	<0.001
Liver disease, *n* (%)	116 (13.7)	69 (17.0)	47 (10.7)	0.008
Alcoholism, *n* (%)	27 (3.2)	16 (3.9)	14 (3.18)	0.551
Mortality, *n* (%)	227 (26.9%)	129 (31.8)	98 (22.3)	0.002
Duration of hospital stay (days), *M* (IQR)	6 (3–10)	7 (4–10)	6 (3–10)	0.090

*n*, number; *μ*, mean; SD, standard deviation; *M*, median; IQR, interquartile range; mmol, millimole; L, litre; g, grams; *μ*mol/L, micromole per litre.

**Table 2:** Predictors associated with in-hospital mortality.

Variable		Odds ratio	95% confidence interval	*P* value
Male gender		1.14	0.84–1.58	0.375
Age greater than 50 yrs		1.05	0.77–1.45	0.720
Hyponatraemia		1.62	1.18–2.22	0.002
Infection		1.01	0.68–1.49	0.932
Malignancies		1.92	0.71–4.93	0.132
Heart failure		1.11	0.69–1.77	0.644
Cranial pathologies		0.87	0.57–1.32	0.498
Chronic kidney disease		1.50	0.92–2.40	0.078
Hyperglycaemia		0.50	0.26–0.87	0.012
Liver disease		1.39	0.89–2.16	0.123
Alcoholism		0.67	0.22–1.72	0.389
Hypoalbuminaemia		2.08	1.46–2.99	<0.001

Hypoalbuminaemia, serum albumin of less than 35 g/L; yrs, years; hyperglycaemia, serum random blood glucose above11.1 mmol/L on admission.

**Table 3:** Multiple logistic regression of the independent variables for mortality.

Variable	Z	Standard error	Odds ratio	95% confidence interval	*P* value
Serum sodium (mmol/L)	−4.14	0.013	0.93	0.92–0.97	<0.001
Serum albumin (g/L)	−2.62	0.013	0.96	0.94–0.99	0.009
Total protein (g/L)	0.19	0.009	1.00	0.98–1.02	0.846
Serum urea (mmol/L)	1.39	0.009	1.01	0.99–1.03	0.164
Serum creatinine (*μ*mol/L)	−0.42	0.002	0.99	0.99–1.00	0.675
Length of hospital stay (days)	−1.21	0.139	0.98	0.96–1.01	0.227

## Data Availability

All data are included within the article.
